# Analysis of Variations in the Glutamate Receptor, N-Methyl D-Aspartate 2A (*GRIN2A*) Gene Reveals Their Relative Importance as Genetic Susceptibility Factors for Heroin Addiction

**DOI:** 10.1371/journal.pone.0070817

**Published:** 2013-08-05

**Authors:** Bin Zhao, Yongsheng Zhu, Wei Wang, Hai-min Cui, Yun-peng Wang, Jiang-hua Lai

**Affiliations:** 1 College of Forensic Science, Xi’an Jiaotong University, Key Laboratory of Ministry of Public Health for Forensic Science, Xi’an, Shaanxi, PR China; 2 Key Laboratory of Environment and Genes Related to Diseases, Xi’an Jiaotong University, Ministry of Education, Xi’an, Shaanxi, PR China; 3 Department of Medical Genetics and Cell Biology, Ningxia Medical University, Key Laboratory of Fertility Preservation and Maintenance, Ningxia Medical University, Ministry of Education, Ningxia, Yinchuan, PR China; University of Texas MD Anderson Cancer Center, United States of America

## Abstract

The glutamate receptor, N-methyl D-aspartate 2A (*GRIN2A*) gene that encodes the 2A subunit of the N-methyl D-aspartate (NMDA) receptor was recently shown to be involved in the development of opiate addiction. Genetic polymorphisms in *GRIN2A* have a plausible role in modulating the risk of heroin addiction. An association of *GRIN2A* single-nucleotide polymorphisms (SNPs) with heroin addiction was found earlier in African Americans. To identify markers that contribute to the genetic susceptibility to heroin addiction, we examined the potential association between heroin addiction and forty polymorphisms of the *GRIN2A* gene using the MassARRAY system and GeneScan in this study. The frequency of the (GT)26 repeats (rs3219790) in the heroin addiction group was significantly higher than that in the control group (*χ^2^* = 5.360, *P* = 0.021). The allele frequencies of three polymorphisms (rs1102972, rs1650420, and rs3104703 in intron 3) were strongly associated with heroin addiction (*P*<0.001, 0.0002, and <0.001, after Bonferroni correction). Three additional SNPs from the same intron (rs1071502, rs6497730, and rs1070487) had nominally significant *P* values for association (*P*<0.05), but did not pass the threshold value. Haplotype analysis revealed that the G-C-T-C-C-T-A (block 6) and T-T (block 10) haplotypes of the *GRIN2A* gene displayed a protective effect (*P* = <0.001 and 0.003). These findings point to a role for *GRIN2A* polymorphisms in heroin addiction among the Han Chinese from Shaanxi province, and may be informative for future genetic or neurobiological studies on heroin addiction.

## Introduction

Heroin addiction is a chronically relapsing disease characterized by compulsive drug seeking, drug abuse, tolerance, and physical dependence. According to an adoption study, substance dependence, in general, and opioid addiction, in particular, has a genetic component [1]. Family and twin studies have consistently demonstrated a substantial genetic influence on the development of drug addiction, with inherited risk estimates in the range of 40–60% [2], [3]. Chronic drug use alters gene expression, which activates or attenuates biochemical pathways and produces neuroadaptive changes in signal transduction functions. Recent studies suggested that polymorphisms in the N-methyl D-aspartate 2A (*GRIN2A*) gene may be associated with drug addiction, including alcohol and heroin addiction [4], [5].

Glutamatergic neurotransmission is the major excitatory system in the human brain, and genes encoding glutamate receptors are candidate targets for treatment of neuropsychiatric disorders. N-methyl D-aspartate (NMDA) receptors are key factors in glutamatergic neurotransmission which are involved in brain development, excitatory neurotransmission, synaptic plasticity, and memory formation [6]–[10]. The NMDA receptors are composed of multiple subunits, including at least one NR1 subunit and one or more NR2 subunits (GRIN2A-D) [11], and less commonly, a NR3 subunit (GRIN3A-B) [12]. *GRIN2A* knockout mice show increased spontaneous locomotor activity and deficits in contextual fear conditioning and spatial learning, along with reduced hippocampal long-term potentiation [13], [14] that is thought to be involved in addiction [15]. Moreover, *GRIN2A* knockout mice failed to show evidence of conditioned place preference, suggesting an impairment in learned reward-related responses to ethanol [16]. Several studies have shown that chronic administration of drugs of abuse, such as alcohol [17], methamphetamine [18], cocaine [19], and nicotine [20], alters the activity of GRIN2A in the brain, suggesting that the *GRIN2A* gene is an excellent candidate target for treatment of addiction disorders. Importantly, these results indicate that glutamatergic transmission, particularly through GRIN2A-containing NMDA receptors in the nucleus accumbens, probably contributes to the development of opiate addiction and confirms the hypothesis that subtype-selective NMDA receptor antagonists may be beneficial in the treatment of opiate addiction and withdrawal [21].

The human *GRIN2A* gene is located on chromosome 16p13.2 and consists of twelve exons and thirteen introns which undergo alternative splicing to form a family of *GRIN2A* isoforms. Itokawa et al. [22] have identified a variable (GT)n repeat polymorphism (rs3219790) in the promoter region of this gene that elicited repression of transcriptional activity in a length-dependent manner. Recently, a case-control study showed evidence of an association between the repeat polymorphism and alcohol dependence, with longer alleles overly represented in patients with alcoholism [5]. Moreover, screenings of single-nucleotide polymorphisms (SNPs) in 130 candidate genes incriminated in heroin addiction have recently identified *GRIN2A* as the most significant candidate, and significantly more C-A-T (rs4587976-rs1071502-rs1366076) haplotypes were found in African American heroin-addicted patients [4]. More studies need to be carried out to determine whether these SNPs modulate the risk of disease by themselves or whether they correlate with other causative SNPs and are repeated in other populations.

We hypothesized that common variants in the *GRIN2A* might contribute significantly to the predisposition to develop heroin addiction. In this study, we investigated forty loci in a Chinese population from Shaanxi province to verify the putative association between *GRIN2A* polymorphisms and heroin addiction.

## Subjects and Methods

### 1 Subjects

A total of 210 unrelated subjects with heroin addiction (mean age of 34.82±7.57, 167 males, 43 females) were recruited from the Methadone Maintenance Treatment (MMT) program of Xi’an Mental Health Center. Participants were daily or nearly daily users of heroin for a minimum of one year prior to assessment. The diagnosis of opioid addiction was based on the Diagnostic and Statistical Manual of Mental Disorders (DSM)-IV criteria, medical history, urine test results, and interview responses. Participants were excluded if they: met DSM-IV criteria for an additional Axis I disorder; had a history of alcohol, cigarette, amphetamine, or other drug addiction according to DSM-IV; were taking other prescribed medications that could affect the central nervous system; had a history of seizures, hematological diseases, or severe liver or kidney impairment. In all, 205 healthy blood donors (mean age of 36.13±6.83, 164 males, 41 females) were recruited at the First Hospital Affiliated to the Medical College of Xi’an Jiaotong University. Subjects who had substance abuse, participated in other studies, or suffered from chronic brain diseases were excluded. From 210 subjects, 198 (94%) were tobacco smokers. From 205 controls, 127 (62%) were tobacco smokers. All participants completed a family history questionnaire and were self-identified as Han Chinese from Shaanxi province for three generations. Participants were excluded from the study if they had a relative in this study, or had a mixed ancestry. Written informed consent was obtained from all participants. The study protocol was approved by the Ethical Committee of the Medical College, Xi’an Jiaotong University.

### 2 Selection of Polymorphisms

**Figure 1:**
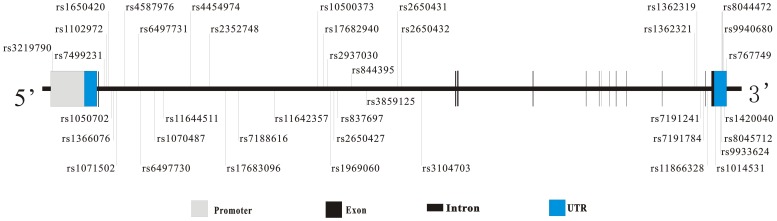
Gene structure of human *GRIN2A* and the relative positions of the 40 variations used in our study.

Polymorphisms in the promoter region, untranslated regions (UTRs), exons, and introns of the gene for glutamate receptor, ionotropic, N-methyl D-aspartate 2A (*GRIN2A*) were systematically screened. Thirty-nine SNPs with minor allele frequencies (MAF) greater than 0.05 were selected from the *GRIN2A* gene and nearby regions based on a review of published literature (Table S1, Table S2) and a search of HapMap and dbSNP (Han Chinese population). The positions of the polymorphisms in the *GRIN2A* gene are shown in Fig. 1 and Table 1.

**Table 1:** Genotype and allele frequencies of the *GRIN2A* gene SNPs in cases and controls and the results of their associations with risk of heroin addiction.

SNP	Variable	Location	Group	Genotype (n, %)			Allele (n, %)		*P* a	*P* b	*P* c	OR^d^, 95%CI^d^
1	rs767749	3′UTR		TT	TG	GG	T	G	0.147	0.578	0.271	1.172, 0.884–1.554
			case	37 (17.6)	89 (42.4)	84 (40.0)	163 (38.8)	257 (61.2)				
			control	30 (14.6)	84 (41.1)	91 (44.4)	144 (35.2)	266 (64.8)				
2	rs1420040	3′UTR		AA	AG	GG	A	G	0.684	0.543	0.263	0.854, 0.647–1.126
			case	70 (33.3)	99 (47.1)	41 (19.6)	239 (56.9)	181 (43.1)				
			control	77 (37.6)	95 (46.3)	33 (16.1)	249 (60.7)	161 (39.3)				
3	rs9940680	3′UTR		GG	GC	CC	G	C	0.960	0.482	0.232	0.845, 0.640–1.114
			case	70 (33.3)	100 (47.6)	40 (19.1)	240 (57.1)	180 (42.2)				
			control	77 (37.6)	97 (47.3)	31 (15.1)	251 (61.2)	159 (38.8)				
4	rs9933624	3′UTR		TT	TC	CC	T	C	0.818	0.536	0.261	0.853, 0.647–1.126
			case	70 (33.3)	100 (47.6)	40 (19.1)	240 (57.1)	180 (42.9)				
			control	77 (37.6)	96 (46.8)	32 (15.6)	250 (61.0)	160 (39.0)				
5	rs8045712	3′UTR		TT	TC	CC	T	C	0.754	0.362	0.181	1.209, 0.916–1.595
			case	40 (19.1)	100 (47.6)	70 (33.3)	180 (42.9)	240 (57.1)				
			control	29 (14.1)	99 (48.3)	77 (37.6)	157 (38.3)	253 (61.7)				
6	rs8044472	3′UTR		GG	GA	AA	G	A	0.566	0.216	0.578	0.923, 0.697–1.223
			case	22 (10.5)	109 (51.9)	79 (37.6)	153 (36.4)	267 (63.6)				
			control	32 (15.6)	93 (45.4)	80 (39.0)	157 (38.3)	253 (61.7)				
7	rs1014531	3′UTR		AA	AG	GG	A	G	0.897	0.688	0.3901	0.864, 0.600–1.21
			case	8 (3.8)	68 (32.4)	134 (63.8)	84 (20.0)	336 (80.0)				
			control	10 (4.9)	72 (35.1)	123 (60.0)	92 (22.4)	318 (77.6)				
8	rs11866328	Intron13		TT	TG	GG	T	G	0.897	0.584	0.302	0.839, 0.619–1.206
			case	8 (3.8)	66 (31.5)	136 (64.7)	82 (19.5)	338 (80.5)				
			control	10 (4.9)	72 (35.1)	123 (60.0)	92 (22.4)	318 (77.6)				
9	rs7191784	Intron12		AA	AG	GG	A	G	0.779	0.835	0.601	1.078, 0.813–1.429
			case	30 (14.3)	100 (47.6)	80 (38.1)	160 (38.1)	260 (61.9)				
			control	28 (13.7)	93 (45.4)	84 (41.0)	149 (36.3)	261 (63.7)				
10	rs7191241	Intron12		CC	CT	TT	C	T	0.720	0.875	0.706	1.056, 0.797–1.398
			case	30 (14.3)	100 (47.6)	80 (38.1)	160 (38.1)	260 (61.9)				
			control	29 (14.1)	93 (45.4)	83 (40.5)	151 (36.8)	259 (63.2)				
11	rs1362319	Intron12		CC	CA	AA	C	A	0.851	0.724	0.622	0.934, 0.711–1.226
			case	57 (27.1)	97 (46.2)	56 (26.7)	211 (50.2)	209 (49.8)				
			control	56 (27.3)	101 (49.3)	48 (23.4)	213 (51.9)	197 (48.1)				
12	rs1362321	Intron12		AA	AG	GG	A	G	0.556	0.946	0.985	1.003, 0.763–1.317
			case	51 (24.3)	97 (46.2)	62 (29.5)	199 (47.4)	221 (52.6)				
			control	48 (23.4)	98 (47.8)	59 (28.8)	194 (47.3)	216 (52.7)				
13	rs3104703	Intron 3		TT	TG	GG	T	G	0.884	0.0003	<0.001	0.576, 0.437–0.758
			case	43 (20.5)	92 (43.8)	75 (35.7)	178 (42.4)	242 (57.6)				
			control	64 (31.2)	102 (49.8)	39 (19.0)	230 (56.1)	180 (43.9)				
14	rs2650432	Intron 3		CC	CT	TT	C	T	0.827	0.524	0.760	0.955, 0.711–1.283
			case	23 (11.0)	80 (38.1)	107 (50.9)	126 (30.0)	294 (70.0)				
			control	19 (9.3)	89 (43.4)	97 (47.3)	127 (31.0)	283 (69.0)				
15	rs2650431	Intron 3		CC	CT	TT	C	T	0.670	0.341	0.289	1.162, 0.880–1.533
			case	41 (19.5)	95 (45.2)	74 (35.3)	177 (42.1)	243 (57.9)				
			control	29 (14.1)	100 (48.8)	76 (37.1)	158 (38.5)	252 (61.5)				
16	rs3859125	Intron 3		CC	CT	TT	C	T	0.616	0.513	0.479	1.110, 0.832–1.481
			case	28 (13.3)	90 (42.9)	92 (43.8)	146 (34.8)	274 (65.2)				
			control	20 (9.8)	93 (45.4)	92 (45.0)	133 (32.4)	277 (67.6)				
17	rs844395	Intron 3		CC	CT	TT	C	T	0.827	0.833	0.317	1.149, 0.875–1.507
			case	56 (26.7)	110 (52.4)	44 (20.9)	222 (52.9)	198 (47.1)				
			control	53 (25.9)	104 (50.7)	48 (23.4)	205 (51.2)	210 (48.8)				
18	rs837697	Intron 3		TT	TG	GG	T	G	0.619	0.358	0.446	1.112, 0.847–1.460
			case	60 (28.6)	96 (45.7)	54 (25.7)	216 (51.4)	204 (48.6)				
			control	47 (22.9)	106 (51.7)	52 (25.4)	200 (48.8)	210 (51.2)				
19	rs2650427	Intron 3		CC	CT	TT	C	T	0.523	0.567	0.341	0.876, 0.667–1.151
			case	43 (20.5)	104 (49.5)	63 (30.0)	190 (45.2)	230 (54.8)				
			control	46 (22.4)	107 (52.2)	52 (25.4)	199 (48.5)	211 (51.5)				
20	rs1969060	Intron 3		TT	TC	CC	T	C	0.443	0.898	0.839	0.972, 0.741–1.276
			case	55 (26.2)	95 (45.2)	60 (28.6)	205 (48.8)	215 (51.2)				
			control	53 (25.9)	97 (47.3)	55 (26.8)	203 (49.5)	207 (50.5)				
21	rs2937030	Intron 3		CC	CT	TT	C	T	0.934	0.595	0.673	0.943, 0.718–1.238
			case	57 (27.1)	96 (45.7)	57 (27.2)	210 (50.0)	210 (50.0)				
			control	54 (26.3)	103 (50.2)	48 (23.5)	211 (51.5)	199 (48.5)				
22	rs17682940	Intron 3		TT	TC	CC	T	C	0.999	0.970	0.835	0.971, 0.735–1.283
			case	31 (14.8)	101 (48.1)	78 (37.1)	163 (38.8)	257 (61.2)				
			control	32 (15.6)	98 (47.8)	75 (36.6)	162 (39.5)	248 (60.5)				
23	rs10500373	Intron 3		CC	CT	TT	C	T	0.976	0.977	0.852	1.028, 0.772–1.368
			case	26 (12.5)	94 (44.7)	90 (42.8)	146 (34.7)	274 (65.3)				
			control	24 (11.7)	92 (44.9)	89 (43.4)	140 (34.1)	270 (65.9)				
24	rs11642357	Intron 3		GG	GA	AA	G	A	0.678	0.484	0.305	1.159, 0.874–1.537
			case	30 (14.3)	103 (49.0)	77 (36.7)	163 (38.8)	257 (61.2)				
			control	27 (13.2)	91 (44.4)	87 (42.4)	145 (35.4)	265 (64.6)				
25	rs7188616	Intron 3		CC	CG	GG	C	G	0.542	0.162	0.807	1.035, 0.787–1.360
			case	36 (17.1)	119 (56.7)	55 (26.2)	191 (45.5)	229 (54.5)				
			control	43 (21.0)	97 (47.3)	65 (31.7)	183 (44.6)	227 (55.4)				
26	rs17683096	Intron 3		AA	AG	GG	A	G	0.283	0.159	0.707	1.054, 0.802–1.385
			case	40 (19.1)	116 (55.2)	54 (25.7)	196 (46.7)	224 (53. 3)				
			control	46 (22.4)	94 (46.3)	65 (31.3)	186 (45.6)	224 (.54.4)				
27	rs2352748	Intron 3		AA	AG	GG	A	G	0.345	0.152	0.516	1.094, 0.833–1.438
			case	44 (20.9)	113 (53.8)	53 (25.3)	201 (47.9)	219 (52.1)				
			control	46 (22.4)	95 (46.3)	64 (31.3)	187 (45.6)	223 (54.4)				
28	rs4454974	Intron 3		CC	CT	TT	C	T	0.566	0.152	0.079	1.281, 0.971–1.690
			case	39 (18.6)	108 (51.4)	63 (30.0)	186 (44.3)	234 (55.7)				
			control	32 (15.6)	93 (45.4)	80 (39.0)	157 (38.3)	253 (61.7)				
29	rs11644511	Intron 3		AA	AC	CC	A	C	0.555	0.994	0.918	0.985, 0.739–1.313
			case	22 (10.5)	98 (46.7)	90 (42.8)	142 (33.8)	278 (66.2)				
			control	22 (10.7)	96 (46.8)	87 (42.5)	140 (34.1)	270 (65.9)				
30	rs1070487	Intron 3		GG	GA	AA	G	A	0.413	0.039	0.041	1.598, 1.017– 2.511
			case	178 (84.8)	29 (13.8)	3 (1.4)	385 (91.7)	35 (8.3)				
			control	155 (75.6)	48 (23.4)	2 (1.0)	358 (87.3)	52 (12.7)				
31	rs6497730	Intron 3		GG	GA	AA	G	A	0.376	0.042	0.042	1.584, 1.013–2.476
			case	177 (84.3)	30 (14.3)	3 (1.4)	384 (91.4)	36 (8.6)				
			control	154 (75.1)	49 (23.9)	2 (1.0)	357 (87.1)	53 (12.9)				
32	rs6497731	Intron 3		GG	GC	CC	G	C	0.623	0.615	0.334	0.874, 0.666–1.148
			case	45 (21.4)	109 (51.9)	56 (26.7)	199 (47.4)	221 (52.6)				
			control	51 (24.9)	106 (51.7)	48 (23.4)	208 (50.7)	202 (49.3)				
33	rs4587976	Intron 3		CC	CG	GG	C	G	0.613	0.282	0.133	0.747, 0.510–1.094
			case	6 (2.9)	44 (20.9)	160 (76.2)	56 (13.3)	364 (86.7)				
			control	7 (3.4)	56 (27.3)	142 (69.3)	70 (17.1)	340 (82.9)				
34	rs1071502	Intron 3		CC	CT	TT	C	T	0.759	0.029	0.007	1.465, 1.109–1.937
			case	43 (20.5)	102 (48.6)	65 (30.9)	188 (44.7)	232 (55.3)				
			control	27 (13.2)	92 (44.9)	86 (41.9)	146 (35.6)	264 (64.4)				
35	rs1366076	Intron 3		AA	AT	TT	A	T	0.506	0.336	0.153	0.819, 0.623–1.077
			case	35 (16.7)	109 (51.9)	66 (31.4)	179 (42.6)	241 (57.4)				
			control	44 (21.5)	107 (52.2)	54 (26.3)	195 (47.6)	215 (52.4)				
36	rs1070502	Intron 3		GG	GT	TT	G	T	0.318	0.431	0.358	0.880, 0.670–1.156
			case	55 (26.2)	103 (49.0)	52 (24.8)	213 (50.7)	207 (49.3)				
			control	56 (27.3)	109 (53.2)	40 (19.5)	221 (53.9)	189 (46.1)				
37	rs1650420	Intron 3		AA	AG	GG	A	G	0.562	0.001	0.0002	1.671, 1.270–2.198
			case	68 (32.4)	103 (49.0)	39 (18.6)	239 (56.9)	181 (43.1)				
			control	42 (20.5)	97 (47.3)	66 (32.2)	181 (44.1)	229 (55.9)				
38	rs1102972	Intron 3		CC	CT	TT	C	T	0.771	<0.001	<0.001	2.143, 1.624–2.828
			case	73 (34.8)	99 (47.2)	38 (18.0)	245 (58.3)	175 (41.7)				
			control	33 (16.1)	96 (46.8)	76 (37.1)	162 (39.5)	248 (60.5)				
39	rs7499321	Intron 3		CC	CT	TT	C	T	0.396	0.327	0.233	1.180, 0.899–1.550
			case	55 (26.2)	102 (48.6)	53 (25.2)	212 (50.5)	208 (49.5)				
			control	41 (20.0)	108 (52.7)	56 (27.3)	190 (46.3)	220 (53.7)				

a values for Hardy-Weinberg equilibrium in controls.

b values for genotype frequency dirrerence.

c values for allele frequency dirrerence.

d values for allele frequency dirrerence.

Alpha value is adjusted by Bonferroni correction and significant results (*P*<0.0013).

### 3 Genotyping

Peripheral blood were collected from the enrolled subjects in tubes coated with EDTA. Genomic DNA was extracted from blood leukocytes using the EZNA™ Blood DNA Midi Kit (Omega Bio-Tek, Norcross, GA, USA) according to the manufacturer’s protocol. Probes and primers were designed using the Assay Design Software (Sequenom, San Diego, CA, USA). SNP genotyping was performed using matrix assisted laser desorption ionization-time of flight (MALDI-TOF; MassARRAY system, Sequenom Inc., San Diego, CA, USA) mass spectrometry. Briefly, one 5-µl PCR reaction include the following reagents: 1 µl of diluted DNA sample, 0.95 µl of water, 0.625 µl of PCR buffer containing 15 mM MgCl_2_, 1 µl of 2.5 mM dNTP, 0.325 µl of 25 mM MgCl_2_, 1 µl of PCR primers and 0.1 µl of 5 units/µl HotStar *Taq* (Qiagen). The reaction was incubated at 94°C for 15 minutes followed by 45 cycles at 94°C for 20 seconds, 56°C for 30 seconds, and 72°C for 1 minute, and a final incubation at 72°C for 3 minutes. The single base extension reaction was carried out at 94°C for 30 seconds and then 94°C for 5 seconds, followed by 5 cycles of 52°C for 5 seconds and 80°C for 5 seconds, total 40 cycles, then 72°C for 3 minutes. The reaction mix was desalted by adding 6 mg of cation exchange resin (Sequenom), mixed and resuspended in 25 µl of water. The completed genotyping reactions were spotted onto a 384 well spectroCHIP (Sequenom) using the MassARRAY Nanodispenser (Sequenom) and determined by the matrix-assisted laser desorption ionization time-of-flight mass spectrometer. Genotype calling was performed in real time with the MassARRAY RT software version 3.0.0.4 and analyzed using the MassARRAY Typer software version 3.4 (Sequenom).

The primers specific for the (GT)n repeat used to amplify the repeat-containing genomic fragment were: a 6-carboxyfluorescein (FAM)-labeled upstream primer, 5′-GAAGGAAGCA TGTGGGAAATGCAG-3′ (the 3′ end is 98 bp upstream of the 5′end of the (GT)n repeat; see GenBank accession No.AF443855), and a non-labeled downstream primer, 5′-gtttcttGCTGG. GTACAGTTATCCCCCT-3′ (the 3′ end is 19 bp downstream of the 3′ end of the (GT)n repeat) [22]. Polymerase chain reaction (PCR) amplification was performed with an initial denaturation at 95°C for 8 min, prior to 10 cycles of denaturation at 95°C for 30 s, annealing at 60°C for 30 s (−1°C per cycle) and extension for 30 s at 72°C, followed by a further 20 cycles of denaturation at 95°C for 30 s, annealing at 55°C for 30 s and extension for 30 s at 72°C, and a final extension at 72°C for 6 min, using AmpliTaq Gold DNA polymerase (Applied Biosystems, Foster City, CA, USA). PCR products were analyzed using an ABI 3730 sequencer equipped with GeneScan software(Applied Biosystems).

### 4 Statistical Analysis

The allele and genotype frequencies for each individual polymorphism were compared, and Hardy-Weinberg equilibrium was evaluated using the Chi-square test. Associations between polymorphisms and heroin addiction were assessed by the Fisher’s exact test or the Pearson Chi-square test. The statistically significance was set at 0.05. Unconditional logistic regression was used to calculate the odds ratio (OR) and 95% confidence interval (CI) in independent association between each locus and the presence of heroin addiction. The Bonferroni correction was used to adjust the test level when multiple comparisons were conducted, and the *P* value was divided by the total number of loci or haplotypes. All statistical analyses were performed using the SPSS 17.0 software (SPSS Inc., Chicago, IL, USA). Haplotype blocks were defined according to the criteria of Gabriel et al. [23], as implemented in Haploview 4.0, to examine if some SNPs significant in the single marker association analysis also exist in the haplotype blocks. Pair-wise linkage disequilibrium (LD) statistics (D’ and r^2^) and haplotype frequency were computed, and haplotype blocks were constructed using Haploview 4.0 [24].

## Results

**Figure 2:**
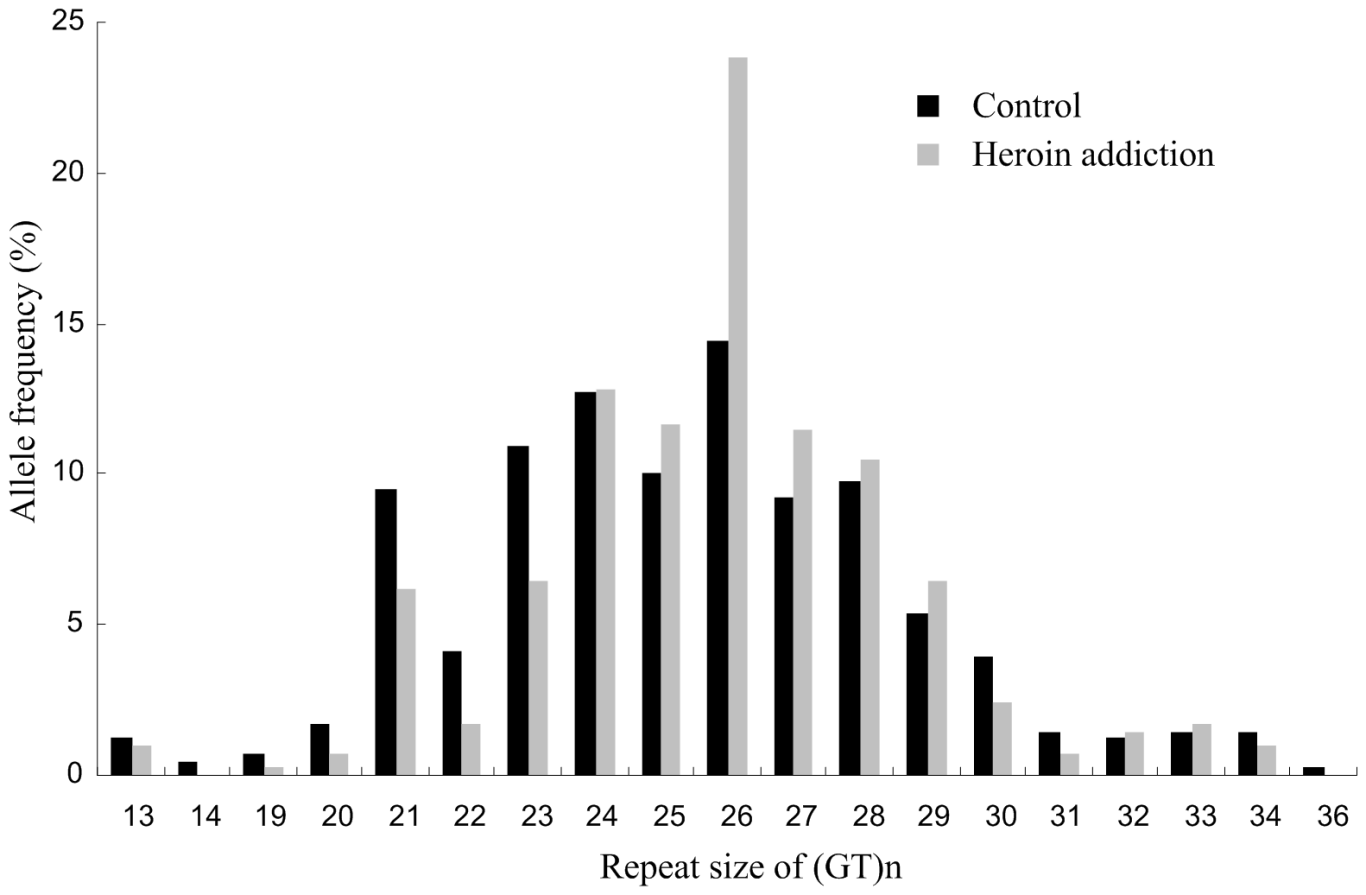
Allele frequency distribution of the *GRIN2A* (GT)n repeat in heroin addiction and controls. **Allele size is expressed as the number of GT repeats.**

We detected repeat numbers ranging from 13 to 36 in the samples in this study. The dominant allele was (GT)26. The allele distribution histogram of heroin-addictedpatients was shifted to the right, with longer alleles over-represented in heroin addiction (Fig. 2). The frequency of the (GT)26 allele in the heroin addiction group was significantly higher than that in the control group (*χ^2^* = 5.360, *P* = 0.021) (Table 2). When we tested for single allelic association of common alleles [frequency >5%: (GT)21 to (GT)29], the Pearson Chi-square test gave the following P values: 0.075 for (GT)21, 0.105 for (GT)22, 0.054 for (GT)23, 0.940 for (GT)24, 0.249 for (GT)25, 0.021 for (GT)26, 0.307 for (GT)27, 0.731 for (GT)28 and 0.516 for (GT)29 (Table 2).

**Table 2:** The statistics of the *GRIN2A* (GT)n repeat in heroin addiction and controls.

(GT)n repeat	Cases (n, %)	Controls (n, %)	χ^2^	*P*
(GT)13	4 (1.0)	5 (1.2)	0.138	0.710
(GT)14	0 (0.0)	2 (0.5)	2.054	0.152
(GT)19	1 (0.2)	3 (0.7)	1.054	0.305
(GT)20	3 (0.7)	7 (1.7)	1.719	0.190
(GT)21	26 (6.2)	39 (9.5)	3.171	0.075
(GT)22	7 (1.7)	17 (4.1)	2.543	0.105
(GT)23	27 (6.4)	45 (11.0)	3.915	0.054
(GT)24	54 (12.9)	52 (12.7)	0.006	0.940
(GT)25	49 (11.7)	41 (10.0)	1.330	0.249
(GT)26	100 (23.8)	59 (14.4)	5.360	0.021
(GT)27	48 (11.4)	38 (9.3)	1.042	0.307
(GT)28	44 (10.5)	40 (9.8)	0.118	0.731
(GT)29	27 (6.4)	22 (5.4)	0.422	0.516
(GT)30	10 (2.4)	16 (3.9)	1.583	0.208
(GT)31	3 (0.7)	6 (1.5)	1.086	0.297
(GT)32	6 (1.4)	5 (1.2)	0.069	0.792
(GT)33	7 (1.7)	6 (1.5)	0.056	0.814
(GT)34	4 (1.0)	6 (1.5)	0.455	0.500
(GT)36	0 (0.0)	1 (0.2)	1.026	0.311

**Figure 3:**
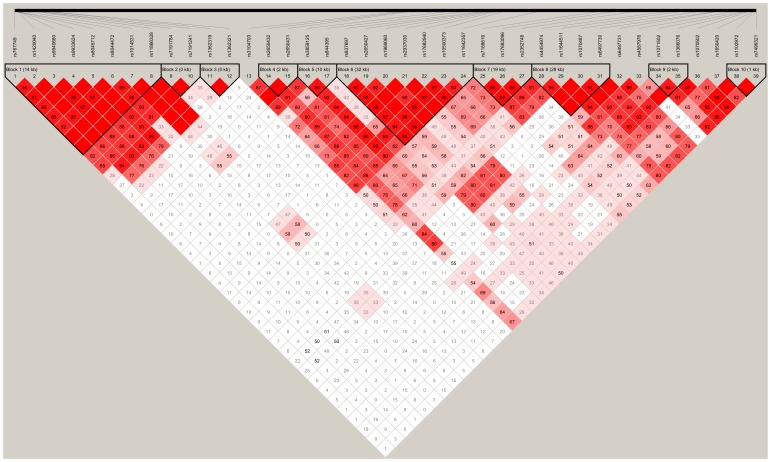
LD plot of the 39 SNPs in *GRIN2A* gene in controls (n = 205). Values in squares are the pair-wise calculation of D’.

**Figure 4:**
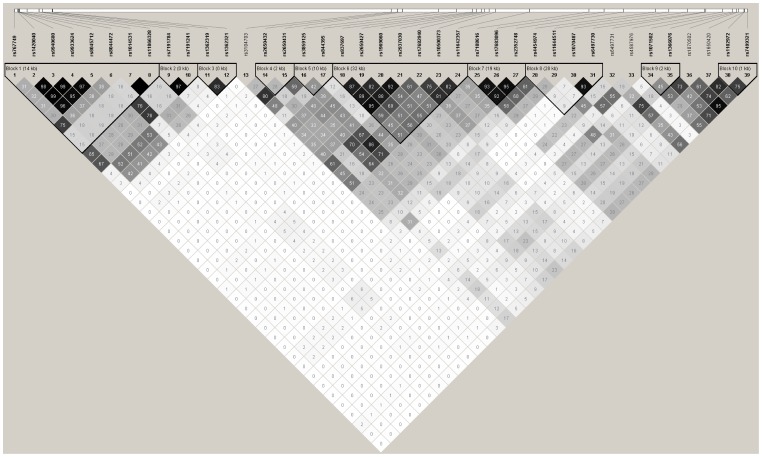
LD plot of the 39 SNPs in *GRIN2A* gene in controls (n = 205). Values in squares are the pair-wise calculation of r^2^.

The distribution frequencies of thirty-nine genotyped SNPs were in agreement with the Hardy-Weinberg equilibrium(*P*>0.05, Table 1). Linkage disequilibrium (LD) analyses data revealed 10 haplotypes in controls, and also showed that 8 SNPs (rs767749, rs1420040, rs9940680, rs9933624, rs8045712, rs8044472, rs1014531, and rs11866328) were located in block 1, 2 SNPs (rs7191784 and rs7191241) in block 2, 2 SNPs (rs1362319 and rs1362321) in block 3, 2 SNPs (rs2650432 and rs2650431) in block 4, 2 SNPs (rs3859125 and rs844395) in block 5, 7 SNPs (rs837697, rs2650427, rs1969060, rs2937030, rs17682940, rs10500373, and rs11642357) in block 6, 3 SNPs (rs7188616, rs17683096, and rs2352748) in block 7, 4 SNPs (rs4454974, rs11644511, rs1070487, and rs6497730) in block 8, 2 SNPs (rs1071502 and rs1366076) in block 9, and 2 SNPs (rs1102972 and rs7499321) in block 10 (Fig. 3 and Fig. 4). The genotype distributions, allelic frequencies, and haplotypes in the patient and control groups, together with the results of statistical analysis are listed in Tables 1 and 3.

**Table 3:** *GRIN2A* haplotype frequencies and the results of their associations with risk of heroin addiction.

Block	SNP	Haplotype^a^	Case (n, %)	Control(n, %)	χ^2^	*P*	OR, 95%CI
1	1/2/3/4/5/6/7/8	G-G-C-C-T-A-G-G	86 (37.1)	79 (38.5)	0.253	0.615	1.106, 0.746–1.639
		T-A-G-T-C-G-G-G	68 (32.4)	70 (34.1)	2.048	0.152	1.338, 0.898–1.993
		G-A-G-T-C-A-A-T	38 (18.1)	46 (22.4)	1.212	0.271	0.764, 0.472–1.235
2	9/10	G-T	127 (60.5)	133 (64.9)	0.859	0.354	0.828, 0.556–1.123
		A-C	78 (37.1)	76 (37.1)	0.000	0.988	1.003, 0.673–1.494
3	11/12	C-A	97 (46.2)	100 (48.8)	0.279	0.597	0.901, 0.613–1.325
		A-G	102 (48.6)	100 (48.8)	0.002	0.966	0.992, 0.675–1.457
		T-C	86 (41.0)	81 (39.5)	0.089	0.765	1.062, 0.717–1.572
4	14/15	C-T	62 (29.5)	65 (31.7)	0.233	0.629	0.902, 0.594–1.370
		T-T	57 (27.1)	64 (31.2)	0.835	0.361	0.821, 0.537–1.254
		C-C	68 (32.4)	67 (32.7)	0.004	0.948	0.986, 0.654–1.487
5	16/17	T-T	94 (44.8)	101 (49.3)	0.846	0.359	0.834, 0.567–1.227
		T-C	40 (19.0)	41 (20.0)	0.060	0.807	0.941, 0.579–1.529
6	18/19/20/21/22/23/24	G-C-T-C-T-C-G	69 (32.9)	67 (32.7)	0.001	0.970	1.008, 0.669–1.519
		T-T-C-T-C-T-A	97 (46.2)	101 (49.3)	0.394	0.530	0.884, 0.601–1.300
		G-C-T-C-C-T-A	137 (65.2)	185 (90.2)	37.305	<0.001	0.203, 0.118–0.349
7	25/26/27	C-A-A	93 (44.3)	92 (44.9)	0.015	0.903	0.976, 0.663–1.438
		G-G-G	106 (50.5)	113 (55.1)	0.898	0.343	0.830, 0.564–1.221
8	28/2930/31	C-C-G-G	88 (41.9)	78 (38.0)	0.643	0.423	1.174, 0.793–1.740
		T-A-G-G	66 (31.4)	71 (34.6)	0.482	0.488	0.865, 0.574–1.303
		T-C-G-G	31 (14.8)	32 (15.6)	0.058	0.810	0.936, 0.548–1.601
		T-C-A-A	16 (7.6)	26 (12.7)	2.924	0.087	0.568, 0.295–1.093
9	34/35	C-T	91 (43.3)	73 (35.6)	2.589	0.108	1.383, 0.931–2.053
		T-A	87 (41.4)	98 (47.8)	1.707	0.191	0.772, 0.524–1.138
		T-T	26 (12.4)	37 (18.0)	2.588	0.108	0.642, 0.373–1.105
10	38/39	C-C	103 (49.0)	83 (40.5)	3.073	0.080	1.415, 0.959–2.087
		T-T	85 (40.5)	113 (55.1)	8.919	0.003	0.554, 0.375–0.817
		T-C	17 (8.1)	14 (6.8)	0.241	0.624	1.202, 0.576–2.506

Haplotypes^a^ with frequency <0.05 were excluded.

The frequency of the T allele in rs1102972 (χ^2^ = 29.408, *P*<0.001, odds ratio [OR] = 2.143, 95% confidence interval [CI] = 1.624–2.828) and the G allele in rs1650420 (χ^2^ = 13.511, *P* = 0.0002, OR = 1.671, 95% CI = 1.270–2.198) in heroin-addicted subjects was significantly lower than that in the controls (Table 1). The rs3104703 G allele frequency in heroin-addicted subjects was significantly higher than that in the controls (χ^2^ = 15.618, *P*<0.001, OR = 0.576, 95% CI = 0.437–0.758) (Table 1). Furthermore, the rs1071502 T, rs6497730 G, and rs1070487 G allele frequency in heroin addicts were higher than that in the controls (*P*<0.05), but did not pass the threshold value. Thirty-three additional *GRIN2A* SNPs gave negative results (Table 1).

The G-C-T-C-C-T-A haplotype in block 6 occurred significantly more frequently (χ*^2^* = 37.305, *P*<0.001, OR = 0.203, 95% CI = 0.118–0.349, protective) and the T-T haplotypes in block 10 occurred more frequently (χ*^2^* = 8.919, *P* = 0.003, OR = 0.554, 95% CI = 0.375–0.817, protective) in controls (Table 3). These differences were retained even after Bonferroni correction.

## Discussion

GRIN2A regulates reward-related associative learning, cognition, memory, and structural and behavioral plasticity in the context of drug addiction [13]–[16], [21], [25], [26], suggesting that GRIN2A acts upon the brain’s reward system, which plays a key role in drug addiction. In the past decade, accumulated evidence indicates that NMDA receptors play a pivotal role in the development of tolerance and physical dependence to opiates [27]–[29]. Our results provide direct evidence that a genetic change in *GRIN2A* is linked to heroin addiction in humans, and extends the list of variants that may affect the development of heroin addiction [4].

A variable (GT)n repeat in the 5′-regulatory region of the *GRIN2A* gene has been identified [22]. It was shown that the repeat sequence repressed transcriptional activity in a length-dependent manner, such that the longer the repeat, the lower the promoter activity. In this case-controlled association study, significant differences were found in the distribution of allele frequencies of (GT)n repeats in the *GRIN2A* gene between heroin-addicted subjects and healthy controls. The frequency of the (GT)26 repeat in heroin addicts was significantly higher than that in the controls. To our knowledge, our study is the first to identify a significant association between (GT)n repeats in the 5′-regulatory region of the *GRIN2A* gene and heroin addiction. Indeed, heroin-addicted patients had overall longer alleles than the control subjects. The (GT)n polymorphism in the promoter of *GRIN2A* has been reported to be associated with schizophrenia and bipolar disorder [22], [30], [31]. Similarly, a previous study showed that longer alleles of (GT)n repeats were significantly more frequent among alcohol dependence [5]. The average observed repeat number distributions were significantly different in alcohol-dependent subjects (GT repeats: n = 24.5) and the control subjects (GT repeats: n = 23.7) [5]. It is possible that the presence of longer (GT)26 repeat results in decreased GRIN2A receptor function in patients with heroin addiction. This finding represents clinical-genetic evidence pointing toward the role of promoter (GT)n polymorphisms in the *GRIN2A* gene in the pathophysiology of heroin addiction.

In this study, we evaluated the association between thirty-nine SNPs that efficiently tag the common variation in the *GRIN2A* gene and heroin addiction. The most intriguing finding of the present study is that three *GRIN2A* variants (rs1102972, rs1650420, and rs3104703), all located within a 32-kb area of intron 3, accounted for some of the strongest signals in the association test. Three additional SNPs from the same intron (rs1071502, rs6497730, and rs1070487) had nominally significant *P* values for association (*P*<0.05), but did not pass the threshold value. *GRIN2A* has also recently been shown to be of the highest relevance in human alcohol dependence, among 10 glutamatergic neurosignaling genes [32], and in heroin addiction, among 6 glutamatergic neurosignaling genes [4]. Previously, Levran et al. [4] confirmed a statistically significant association between 6 polymorphisms located at intron 3 (rs1070487, rs6497730, rs4587976, rs1650420, rs1071502, and rs1366076) and heroin-addiction in African Americans. Differences of this kind may be correlated with alterations in hormone levels, neuronal system adaptations, and the pharmacokinetics of substances of abuse [33].

Population stratification is an important issue to be considered when conducting human genetic surveys [34]. In the present study, the experimental and control groups were matched for ethnicity by enrolling subjects from a homogeneous population. Our analysis also has sufficient statistical power to argue an epidemiologically-relevant impact of hereditary variations among the studied genes.

We further investigated the interaction among polymorphisms and observed strong linkage disequilibrium. Haplotype analysis revealed that the G-C-T-C-C-T-A (block 6) and T-T (block 10) haplotypes of the *GRIN2A* gene displayed a protective effect. There were significant point-wise associations of these variants with heroin addiction. These results indicated that people with these two haplotypes of the *GRIN2A* gene were less prone to heroin addiction. A previous haplotype analysis of rs4587976-rs1071502-rs1366076 revealed significant association of the G-A-T (protective) and C-A-T (risk) haplotypes in heroin-dependent patients and healthy controls, respectively [4]. To some extent, this finding further supports a role of *GRIN2A* polymorphisms in heroin addiction, with differences in the specifics of the association between ethnic groups.

In conclusion, these findings encourage future efforts aimed at identifying functional polymorphisms within, and close to, the *GRIN2A* gene using a systemic approach in a larger sample set. Our results are in line with the glutamatergic hypothesis developed to understand the acute and chronic effects of heroin on the brain. The results of this and similar future studies could help understand the neurobiological mechanisms of heroin addiction better, allowing us to devise better treatment strategies.

## Supporting Information

Table S1 **Summary of published psychiatric disorder-association studies for** ***GRIN2A.***
(DOC)

Table S2 **Comparison of the MAF of 39 SNPs between African American (AA) and Chinese Han (CH) population. It should list the source of this information.**
(DOC)
