# Supplementary material for: Analysis of Variations in the Glutamate Receptor, N-Methyl D-Aspartate 2A (GRIN2A) Gene Reveals Their Relative Importance as Genetic Susceptibility Factors for Heroin Addiction
Source: PLoS One. 2013 Aug 5;8(8):e70817. doi: 10.1371/journal.pone.0070817 (PMC3733659; doi:10.1371/journal.pone.0070817)
Supplement: Table S1 — Summary of published psychiatric disorder-association studies for GRIN2A. (DOC) [file pone.0070817.s001.doc]

***Table S1*** *Summary of published psychiatric disorder-association studies for GRIN2A*

| Study | Population | | Type of Study | Sample Size (n) | | Number of  SNPs typed | Positive SNPs |
| --- | --- | --- | --- | --- | --- | --- | --- |
|  | Ethnic group | Country |  | Case | Control |  |  |
| Itokawa et, 2003 ^[22]^ | Mongoloid | Japan | Family-based | 96 families | | 1 | rs3219790 |
| Domart MC, 2012 ^[5]^ | Caucasian | France | Case-control | 168 | 206 | 1 | rs3219790 |
| Levran O, 2009 ^[4]^ | African Americans | USA | Case-control | 202 | 167 | 1350 | rs1650420, rs4587976, rs6497730, rs1070487 |
