# Supplementary material for: Analysis of Variations in the Glutamate Receptor, N-Methyl D-Aspartate 2A (GRIN2A) Gene Reveals Their Relative Importance as Genetic Susceptibility Factors for Heroin Addiction
Source: PLoS One. 2013 Aug 5;8(8):e70817. doi: 10.1371/journal.pone.0070817 (PMC3733659; doi:10.1371/journal.pone.0070817)
Supplement: Table S2 — Comparison of the MAF of 39 SNPs between African American (AA) and Chinese Han (CH) population. It should list the source of this information. (DOC) [file pone.0070817.s002.doc]

**Table S2** Comparison of the MAF of 39 SNPs between African American (AA) and Chinese Han (CH) population. It should list the source of this information.

| SNP | Variable | Location | AA | CH | SNP | Variable | Location | AA | CH |
| --- | --- | --- | --- | --- | --- | --- | --- | --- | --- |
| 1 | rs767749 | 3'UTR | 0.429 | 0.389 | 21 | rs2937030 | Intron 3 | 0.283 | 0.463 |
| 2 | rs1420040 | 3'UTR | 0.478 | 0.415 | 22 | rs17682940 | Intron 3 | 0.152 | 0.326 |
| 3 | rs9940680 | 3'UTR | 0.478 | 0.415 | 23 | rs10500373 | Intron 3 | 0.130 | 0.314 |
| 4 | rs9933624 | 3'UTR | 0.398 | 0.415 | 24 | rs11642357 | Intron 3 | 0.102 | 0.314 |
| 5 | rs8045712 | 3'UTR | 0.281 | 0.415 | 25 | rs7188616 | Intron 3 | 0.143 | 0.463 |
| 6 | rs8044472 | 3'UTR | 0.114 | 0.378 | 26 | rs17683096 | Intron 3 | 0.152 | 0.453 |
| 7 | rs1014531 | 3'UTR | 0.283 | 0.207 | 27 | rs2352748 | Intron 3 | 0.304 | 0.465 |
| 8 | rs11866328 | Intron13 | 0.327 | 0.195 | 28 | rs4454974 | Intron 3 | 0.082 | 0.430 |
| 9 | rs7191784 | Intron12 | 0.167 | 0.378 | 29 | rs11644511 | Intron 3 | 0.041 | 0.372 |
| 10 | rs7191241 | Intron12 | 0.225 | 0.411 | 30 | rs1070487 | Intron 3 | 0.391 | 0.140 |
| 11 | rs1362319 | Intron12 | 0.224 | 0.463 | 31 | rs6497730 | Intron 3 | 0.478 | 0.133 |
| 12 | rs1362321 | Intron12 | 0.214 | 0.476 | 32 | rs6497731 | Intron 3 | 0.102 | 0.488 |
| 13 | rs3104703 | Intron 3 | 0.348 | 0.463 | 33 | rs4587976 | Intron 3 | 0.358 | 0.144 |
| 14 | rs2650432 | Intron 3 | 0.020 | 0.341 | 34 | rs1071502 | Intron 3 | 0.196 | 0.453 |
| 15 | rs2650431 | Intron 3 | 0.204 | 0.400 | 35 | rs1366076 | Intron 3 | 0.175 | 0.411 |
| 16 | rs3859125 | Intron 3 | 0.184 | 0.354 | 36 | rs1070502 | Intron 3 | 0.391 | 0.430 |
| 17 | rs844395 | Intron 3 | 0.333 | 0.475 | 37 | rs1650420 | Intron 3 | 0.326 | 0.444 |
| 18 | rs837697 | Intron 3 | 0.348 | 0.442 | 38 | rs1102972 | Intron 3 | 0.429 | 0.427 |
| 19 | rs2650427 | Intron 3 | 0.389 | 0.433 | 39 | rs7499321 | Intron 3 | 0.429 | 0.439 |
| 20 | rs1969060 | Intron 3 | 0.413 | 0.444 |  |  |  |  |  |

The data of African American (AA) originate from dbSNP HapMap YRI or ASW and the data of Chinese Han (CH) are from dbSNP HapMap CHB.
